# SARS-CoV-2 Spike Antagonizes Innate Antiviral Immunity by Targeting Interferon Regulatory Factor 3

**DOI:** 10.3389/fcimb.2021.789462

**Published:** 2022-01-10

**Authors:** Raul S. Freitas, Tyler F. Crum, Kislay Parvatiyar

**Affiliations:** Department of Microbiology & Immunology, Tulane University School of Medicine, New Orleans, LA, United States

**Keywords:** SARS-CoV-2, IRF3, interferon, RIG-I antiviral, innate immunity

## Abstract

Corona virus disease 2019 (COVID-19) pathogenesis is intimately linked to the severe acute respiratory syndrome corona virus 2 (SARS-CoV-2) and disease severity has been associated with compromised induction of type I interferon (IFN-I) cytokines which coordinate the innate immune response to virus infections. Here we identified the SARS-CoV-2 encoded protein, Spike, as an inhibitor of IFN-I that antagonizes viral RNA pattern recognition receptor RIG-I signaling. Ectopic expression of SARS-CoV-2 Spike blocked RIG-I mediated activation of IFNβ and downstream induction of interferon stimulated genes. Consequently, SARS-CoV-2 Spike expressing cells harbored increased RNA viral burden compared to control cells. Co-immunoprecipitation experiments revealed SARS-CoV-2 Spike associated with interferon regulatory factor 3 (IRF3), a key transcription factor that governs IFN-I activation. Co-expression analysis *via* immunoassays further indicated Spike specifically suppressed IRF3 expression as NF-κB and STAT1 transcription factor levels remained intact. Further biochemical experiments uncovered SARS-CoV-2 Spike potentiated proteasomal degradation of IRF3, implicating a novel mechanism by which SARS-CoV-2 evades the host innate antiviral immune response to facilitate COVID-19 pathogenesis.

## Introduction

Severe acute respiratory syndrome Corona virus 2 (SARS-CoV-2) is a newly emerged pathogen and is associated with Corona virus disease 19 (COVID-19) which can manifest into life threatening upper respiratory pathologies and lung dysfunctions in severely affected individuals (V’kovski et al., 2021). According to the World Health Organization, greater than 257 million people have been infected with SARS-CoV-2 worldwide resulting in more than 5.1 million deaths. SARS-CoV-2 is an enveloped, single stranded, positive sense RNA virus with a genome of nearly 30 kb that is about 79% similar to SARS-CoV and 50% similar to Middle east respiratory syndrome Corona virus (MERS-CoV) (Lu et al., 2020). The SARS-CoV-2 genome contains 23 open reading frames (ORF) encoding several accessory and non-structural proteins (NSP) as well as four conserved structural proteins: Spike, Envelope, Membrane, and Nucleocapsid (Finkel et al., 2021; Redondo et al., 2021).

Innate immunity provides a first line of defense against invading pathogens including viruses in which the induction of type I interferon (IFN-I) cytokines (IFNα/β) plays an essential role in limiting virus replication and spread (Gonzales-van Horn and Farrar, 2015; Ghosh et al., 2016). While IFN-I is regulated at the level of transcription, primarily by the interferon regulatory factor 3 (IRF3) transcription factor, IRF3 activation itself requires engagement of upstream pattern recognition receptors (PRRs) in cells of the innate immune system. In the context of RNA virus infections, viral RNA species are detected by the retinoic induced gene-I (RIG-I) like PRRs situated in the cytosolic compartment (Akira et al., 2006; Pandey et al., 2014; Roers et al., 2016). RIG-I receptor ligation results in its association with the mitochondrial antiviral signaling adaptor, MAVS, which subsequently recruits TNF receptor associated factor 3 (TRAF3) and TRAF6. TRAF3 serves as a scaffold in facilitating complex formations with the viral activated kinases, TANK binding kinase 1 (TBK1) and/or I kappa B kinase epsilon/inducible (IKKi), which activate IRF3 *via* c-terminal phosphorylation to induce the IFN-I response. Alternatively, TRAF6 activates IKKα/β as part of the classical NF-ĸB pathway, leading to the induction of pro-inflammatory cytokines (Liu et al., 2013; Fang et al., 2017). IFN-I elicits an antiviral state by binding to the IFNα/β receptor (IFNAR) on neighboring cells. IFNAR ligation results in the activation of a Janus kinase/Signal transducer and activator of transcription (JAK/STAT) pathway that culminates in the formation of a STAT1/STAT2/IRF9 heterotrimeric transcription factor which binds to IFN stimulated response elements (ISRE) found on the promoters and enhancers of IFN stimulated genes (ISGs). ISGs operate as effectors and restriction factors that directly or indirectly target the virus or at various stages of its replication cycle (Akira et al., 2006; Pandey et al., 2014).

Many viruses that have co-evolved with their hosts have developed complex and multiple strategies to subvert or antagonize the host innate immune response as a mechanism to evade immune detection and elimination. Indeed, it has become increasingly clear that multiple SARS-CoV-2 encoded genes products target innate immune signaling pathways at various levels (Xia et al., 2020; Fung et al., 2021; Han et al., 2021; Kumar et al., 2021; Sui et al., 2021a; Vazquez et al., 2021; Zhao et al., 2021). Here we identify the Spike protein of SARS-CoV-2 as a key negative regulator of IFN-I activation in the RIG-I pathway that targets the IRF3 transcription factor and mediates its proteasomal degradation. Our findings provide key insight as to how SARS-CoV-2 may subvert innate antiviral immunity to instigate disease pathogenesis.

## Results

### SARS-CoV-2 Spike Restricts IFN-I Activation

**Figure 1:**
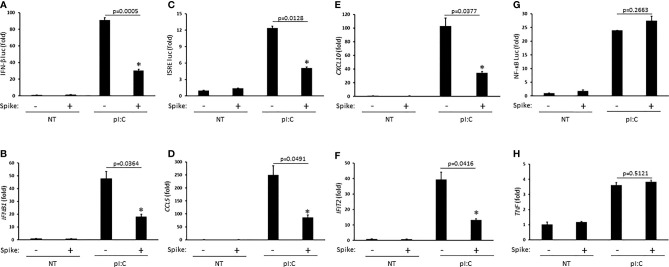
SARS-CoV-2 Spike inhibits pI:C dependent activation of interferon. **(A)** IFN-β luciferase reporter assay in HEK 293T cells co-transfected with pI:C (1 μg) and C9 Spike (1 μg). **(B)** Gene expression levels of *IFNb1* determined *via* Q-PCR in HEK 293T cells co-transfected with pI:C and C9 Spike as in **(A)**. **(C)** ISRE luciferase reporter assay in HEK 293T cells co-transfected with pI:C and C9 Spike as in **(A)**. **(D–F)** Gene expression levels of *CCL5*, *CXCL10*, and *IFIT2* determined *via* Q-PCR in HEK 293T cells as in **(B)**. **(G)** NF-ĸB luciferase reporter assay in HEK 293T cells co-transfected with pI:C and C9 Spike as in **(A)**. **(H)** Gene expression levels of *TNF* determined *via* Q-PCR in HEK 293T cells co-transfected with pI:C and C9 Spike as in **(B)** Data are means ± SEM of one experiment run in triplicate or duplicate out of 2-3 independent experiments. *Indicates p < 0.05 as determined by the *Student’s* t-test.

Innate antiviral immunity against RNA virus infections including SARS-CoV-2 requires activation of the RIG-I pathway (Kouwaki et al., 2021; Yamada et al., 2021). To determine whether SARS-CoV-2 Spike modulated this pathway, HEK 293T cells were first transfected with the synthetic RIG-I ligand, poly (I:C), in the presence or absence of Spike (alpha variant, B.1.1.7). Poly (I:C) stimulation triggered the robust activation of IFNβ which was significantly blunted by Spike as determined by IFN-β luciferase reporter assays and quantitative PCR (Q-PCR) (**Figures 1A, B**). As poly (I:C) mediated activation of IFNβ was suppressed by SARS-CoV-2 Spike, downstream activation of the IFN stimulated response element (ISRE) luciferase reporter and expression of IFN stimulated genes (ISGs) CCL5 (RANTES), CXCL10 (IP-10), and IFIT2 (ISG54) were consequently inhibited by Spike as well (**Figures 1C–F**). Alternatively, activation of the pro-inflammatory NF-ĸB pathway was not affected by SARS-CoV-2 Spike (**Figures 1G–H**). These data suggest SARS-CoV-2 Spike restricts RIG-I mediated innate antiviral immunity by specifically targeting the IFN-I response and not pro-inflammatory signaling.

### SARS-CoV-2 Spike Supports RNA Virus Infection

**Figure 2:**
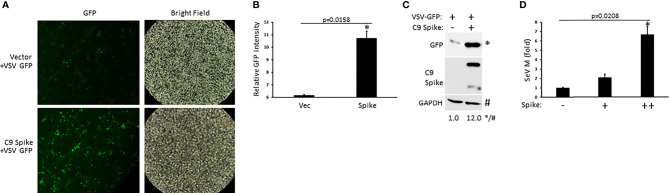
SARS-CoV-2 Spike promotes higher RNA viral loads. **(A)** Fluorescent imaging of HEK 293T cells transfected with control vector or C9 Spike (2 µg) (left panels) and subsequently infected with vesicular stomatitis virus (VSV)-GFP (MOI = 0.01) for 16 h. Right panels, bright field image. **(B)** Relative quantification of GFP intensity from A using ImageJ software. **(C)** Immunoblot analysis of VSV-GFP in HEK 293T cells transfected with control vector or C9 Spike (2 µg) and infected with VSV-GFP (MOI = 0.01) for 16 h. GAPDH serves as a loading control. **(D)** Expression levels of Sendai virus (SeV) M gene determined via Q-PCR in HEK 293T cells transfected with C9 Spike (0.25 and 1 µg) and subsequently infected with SeV (MOI = 0.01) for 16 h. *Indicates p < 0.05 as determined by the student’s t-test. Results are representative of two independent experiments. The relative band intensity (*/#) of GFP in **(C)** was measured using ImageJ software.

Our data indicated the Spike protein from SARS-CoV-2 blocked IFN-I induction in the RIG-I pathway, a critical PRR system that detects RNA viral genomes to instigate the innate antiviral immune response. To determine the physiological consequence of Spike inhibition of IFN-I, HEK 293T cells were transfected with or without SARS-CoV-2 Spike followed by infection with the RNA viruses, vesicular stomatitis virus expressing a green fluorescent protein tag (VSV-GFP) or Sendai virus (SeV). Spike transfected cells displayed greater viral loads compared to cells transfected with a control plasmid as determined *via* live fluorescent imaging and immunoblot analysis for VSV-GFP (**Figures 2A–C**) or *via* quantification of the SeV encoded matrix gene, M (**Figure 2D**). These findings indicate SARS-CoV-2 Spike dampening of IFN-I yields a heightened susceptibility to viral infections.

### SARS-CoV-2 Spike Suppresses RIG-I Mediated Activation of IFN-I at the Level of IRF3

**Figure 3:**
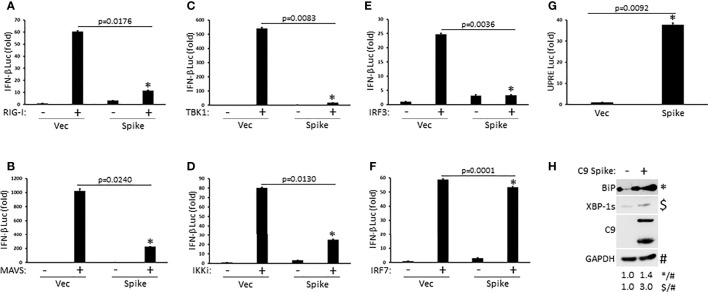
SARS-CoV-2 Spike suppresses RIG-I mediated activation of IFN-I at the level of IRF3. **(A–F)** IFN-β luciferase reporter assays in HEK 293T cells co-transfected with plasmids encoding RIG-I **(A)**, MAVS **(B)**, TBK1 **(C)**, IKKi **(D)**, IRF3 **(E)**, IRF7 **(F)** (1 µg each) and C9 Spike (2 µg). **(G)** UPRE luciferase reporter assay in HEK 293T cells transfected with C9 Spike (2 µg). Data are means ± SEM of one experiment run in duplicate out of 2-3 independent experiments. *Indicates p < 0.05 as determined by the student’s t-test. **(H)** Immunoblot analysis of BiP and XBP-1s in HEK 293T cells transfected with C9 Spike (2 µg). GAPDH serves as a loading control. Results are representative of two independent experiments. The relative band intensity (*/#) of BiP and ($/#) XBP-1s in **(H)** was measured using ImageJ software.

To determine the molecular order at which SARS-CoV-2 Spike inhibited IFN-I activation, HEK 293T cells were co-transfected with plasmids encoding individual signaling components of the RIG-I pathway in the presence or absence of Spike along with a reporter construct for IFN-β luciferase. SARS-CoV-2 Spike significantly blocked IFN-β luciferase activity mediated by the RIG-I PRR itself, the MAVS adaptor, the TBK1 and IKKi kinases, and the IRF3 transcription factor (**Figures 3A–E**). Interestingly, Spike did not dramatically suppress IFN-β reporter activation facilitated by IRF7, a transcription factor with known overlapping functions with IRF3 (**Figure 3F**). These data suggest that SARS-CoV-2 Spike likely inhibits RIG-I activation of IFN-I at the level of the IRF3 transcription factor. In addition to eliciting an IFN-I antiviral response, virus infections can further cause replication stress resulting in the aggregation of unfolded and misfolded proteins in the endoplasmic reticulum (ER) and the triggering of the highly conserved unfolded protein response (UPR) pathway that governs cellular homeostasis (Movaqar et al., 2021; Siri et al., 2021). While SARS-CoV-2 Spike blocked the IFN-I response, it instead potentiated the UPR as demonstrated *via* increased activation of the unfold protein response element as shown *via* luciferase reporter assays (**Figure 3G**). Accordingly, immunoblot analysis in HEK 293T cells transfected with Spike indicated increased expression of the ER chaperone, BiP (aka GRP78), and the splicing dependent activation of XBP-1, which transcriptionally up-regulates genes involved in protein folding as well as ER associated degradation (**Figure 3H**). Taken together, these results find that SARS-CoV-2 Spike has specificity in blocking select immune pathways triggered by virus infections rather than global inhibition.

### SARS-CoV-2 Spike Interacts With IRF3 and Mediates Its Proteasomal Degradation

**Figure 4:**
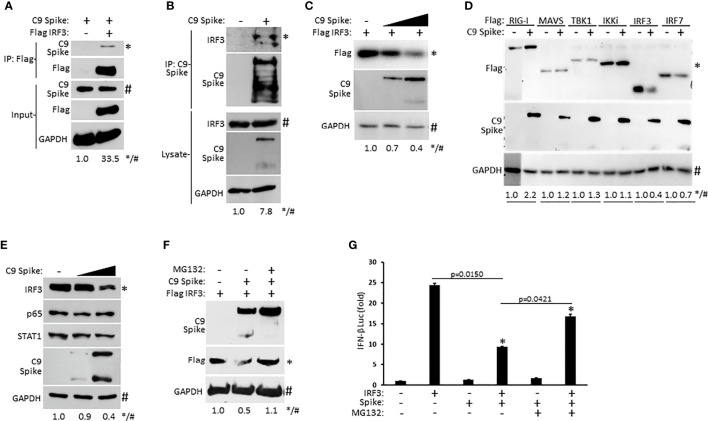
SARS-CoV-2 Spike interacts with IRF3 and mediates its proteasomal degradation. **(A)** Co-immunoprecipitation and immunoblot of Flag IRF3 (1 µg) with C9 Spike (1 µg) co-transfected in HEK 293T cells. **(B)** Immunoprecipitation and immunoblot of transfected C9 Spike (1 µg) with IRF3 in HEK 293T cells treated with MG132 (10 µM) for 4h. **(C)** Immunoblot analysis of Flag IRF3 (250 ng) co-transfected with C9 Spike (500 ng and 1 μg) in HEK293T cells. **(D)** Immunoblot analysis of Flag RIG-I, Flag MAVS, Flag TBK1, Flag IKKi, Flag IRF3, and Flag IRF7 (1 µg each) co-transfected with C9 Spike (2 µg) in HEK 293T cells. **(E)** Immunoblot analysis of IRF3, p65, and STAT1 in HEK 293T cells transfected with C9 Spike (500 ng and 1 µg). **(F)** Immunoblot analysis of Flag IRF3 (1 µg) co-transfected with C9 Spike (2 µg) in HEK 293T cells and subsequently treated with or without MG132 (10 µM). GAPDH serves as a loading control. **(G)** IFN-β luciferase reporter assays in HEK 293T cells co-transfected with plasmids encoding IRF3 (500 ng) and C9 Spike (1 µg) followed by MG132 (20 µM) treatment for 4 h. *Indicates p < 0.05 as determined by the student’s t-test. Results are representative of two independent experiments. The relative band intensity (*/#) of co-immunoprecipitated Flag IRF3 in **(A)**, co-immunoprecipitated endogenous IRF3 in **(B)**, Flag IRF3 in **(C, F)**, Flag RIG-I, Flag MAVS, Flag TBK1, Flag IKKi, Flag IRF3 and Flag IRF7 in **(D)** and endogenous IRF3 in **(E)** was measured using ImageJ software.

As our IFN-β luciferase reporter data above revealed that SARS-CoV-2 Spike restricted RIG-I mediated activation of IFN-I at the level of IRF3, we sought to determine if Spike associated with IRF3. Whole cell lysates from HEK 293T cells co-transfected with epitope tagged plasmids encoding IRF3 and SARS-CoV-2 Spike were immunoprecipitated for IRF3. Subsequent immunoblot analysis indicated immunoprecipitated IRF3 interacted with Spike (**Figure 4A**). Similarly, ectopically expressed Spike in HEK 293T cells was found to associate with endogenous IRF3 *via* immunoprecipitation immunoblot analysis (**Figure 4B**). Since SARS-CoV-2 Spike targeted IRF3, we next asked whether Spike affected the expression of IRF3. Indeed, HEK 293T cells co-transfected with equal quantities of IRF3 along with increasing amounts of Spike displayed a dose dependent decrease in IRF3 expression (**Figure 4C**). Notably, SARS-CoV-2 Spike suppressed the co-expression of IRF3 but not other overexpressed components of the RIG-I pathway that facilitate IFN-I activation (**Figure 4D**) previously shown in **Figures 3A–F**. Furthermore, SARS-CoV-2 Spike specifically decreased IRF3 at the endogenous level but did not alter the expression of the NF-ĸB (p65), and STAT1 transcription factors in HEK 293T cells (**Figure 4E**). To determine how SARS-CoV-2 Spike inhibited IRF3 protein expression, we hypothesized a mechanism involving proteasomal degradation. To this end, HEK 293T cells were co-transfected with IRF3 and Spike in the presence or absence of the proteasomal inhibitor MG132. Immunoblot analysis revealed that IRF3 suppression mediated by Spike could be reversed upon proteasomal inhibition (**Figure 4F**). Likewise, Spike suppression of IRF3 mediated IFN-β reporter activity was partially restored in the presence of the proteasomal inhibitor (**Figure 4G**). Collectively, our data indicate SARS-CoV-2 Spike targets IRF3 and inhibits its function *via* promoting its proteasomal degradation.

## Discussion

SARS-CoV-2 Spike is a large type I transmembrane fusion protein that is heavily glycosylated and plays a key role in facilitating virus infection compared to the other surface proteins, membrane and envelope, found on Coronaviruses. SARS-CoV-2 Spike binds to the angiotensin-converting enzyme 2 (ACE2) receptor on host cells and in conjunction with the transmembrane protease serine 2 (TMPRSS2) found on the host cell membrane, mediates viral entry into the cell (Huang et al., 2020; V’kovski et al., 2021). Upon viral entry, viral RNA is released, allowing for polyprotein translation as well as genome replication and transcription events yielding viral assembly and packaging (Hartenian et al., 2020). Viral RNA is further detected by germ-line encoded PRRs including RIG-I, which triggers the activation of the innate antiviral immune response coordinated by IFN-I. These cytokines activate an antiviral program resulting in the induction of nearly 300 ISGs, which collectively foster an antiviral state leading to viral inhibition and spread. Here we identified SARS-CoV-2 Spike as a key inhibitor of IFN-I in the RIG-I PRR pathway. While Spike suppressed the IFN-I response upon RIG-I stimulation and the downstream induction of ISGs, it did not block NF-ĸB and pro-inflammatory cytokine activation normally conferred during RIG-I signaling. Additional reports indicate the RIG-I like receptor, melanoma differentiation associated gene 5 (MDA5) can also detect SARS-CoV-2 genomic RNA to trigger the host IFN-I response in human lung epithelial cells (Rebendenne et al., 2021; Sampaio et al., 2021; Yin et al., 2021). As MDA5 utilizes the same adaptor (MAVS), kinases (TBK1/IKKi), and transcription factor (IRF3) as RIG-I to activate IFN-I, it may be likely SARS-CoV-2 Spike suppresses MDA5 signaling *via* a similar mechanism. SARS-CoV-2 encodes a variety of other genes that antagonize innate immunity, suggesting SARS-CoV-2 employs a network of genes to collectively circumvent innate immune activation (Lei et al., 2020; Beyer and Forero, 2021).

In contrast to blocking the innate IFN-I response, we found Spike elevated a separate viral triggered cell stress pathway (UPR), suggesting that SARS-CoV-2 Spike does not globally antagonize broad antiviral programs. As the UPR pathway can initiate autophagic or cell death outcomes, it will be of future interest to determine the overall impact of SARS-CoV-2 Spike activation of this pathway and its impact on COV1D-19 pathology.

Consistent with our findings, it was recently reported that recombinant SARS-CoV-2 Spike protein, when incubated with primary cells from lung bronchoalveolar lavage from rhesus macaques, was able to suppress IFN-I levels (Sui et al., 2021b). Our data, in part, attempts to uncover the mechanism by which Spike suppresses the IFN-I response by revealing that SARS-CoV-2 Spike targets the IRF3 transcription factor. Recent studies found SARS-CoV-2 NSP6 inhibited IRF3 by suppressing its phosphorylation mediated by the upstream kinase TBK1 (Xia et al., 2020). Additionally, SARS-CoV-2 ORF6 was reported to limit IRF3 signaling by targeting host factors that facilitate IRF3 translocation into the nuclear compartment where it binds the IFNβ promoter after phosphorylation dependent activation (Xia et al., 2020). Similarly, SARS-CoV-2 NSP12 was shown to block IRF3 nuclear translocation, however a new report found NSP12 was incapable of inhibiting IFNβ activation (Li et al., 2021; Wang et al., 2021).

We found SARS-CoV-2 Spike interacted with IRF3 and further instigated its proteasomal mediated degradation to terminate IFN-I activation. SARS-CoV-2 Spike is comprised of an S1 and S2 domain where SARS-CoV-2 entry requires proteolytic cleavage at these sites (Huang et al., 2020). In many of our immunoblot experiments that examine ectopic Spike expression, we noted the detection of both the full length Spike as well as the faster migrating, but lower intensity S2 domain. However, in some of our immunoblots, (particularly relating to Spike-IRF3 characterizations, **Figures 4A, D**), while the full- length Spike was detected, the S2 domain was below detection capabilities. This may possibly be due to technical circumstances in our experimental design where we reduced cell harvest times post Spike transient transfections in order to minimize excessive or complete IRF3 suppression. Nevertheless, our data suggests that proteasomal inhibition countered the inhibitory effect of Spike on IRF3 in terms of expression and IFN-I activation. Interestingly, we found proteasomal inhibition to not only restore IRF3 expression in the presence of Spike, but also to increase Spike expression and minimize its proteolytic cleavage (**Figure 4F**). Thus, it may be possible that SARS-CoV-2 Spike is subjected to host cell mediated proteasomal degradation. A recent study identified the 3C-like protease of SARS-CoV-2 to also inhibit IRF3 in a similar fashion, while another report indicated it cleaves IRF3 (Shin et al., 2020; Moustaqil et al., 2021; Zhang et al., 2021). Thus, additional studies will be needed to clarify exactly how the 3C-like protease inhibits IRF3. Nevertheless, if this protease indeed is involved in the proteasomal degradation of IRF3, it is tempting to speculate 3C-like protease and Spike may be operating together to subvert IFN-I activation. It is also noteworthy that Spike (nor 3C-like protease) is not known to confer E3 ubiquitin ligase function. As multiple E3 ligases have been described in fostering the degradation of IRF3, further investigation will be required to determine which E3(s) and potential co-factors cooperate with Spike to degrade IRF3 (Higgs et al., 2008; Zhang et al., 2008; Yu and Hayward, 2010; Lei et al., 2013; Wang et al., 2015, Chen et al., 2021).

Taken altogether, our work identified a novel mechanism by which the Spike protein of SARS-CoV-2 operates beyond its currently understood role in host cell attachment and entry. Accordingly, we found Spike antagonized the host IFN-I antiviral innate immune response in the RIG-I signaling pathway by targeting IRF3 for degradation. These findings shed new light into the potential mechanisms of SARS-CoV-2 pathophysiology and magnify the importance of Spike as a key target for therapeutic intervention against COVID-19.

## Materials and Methods

### Cell Culture, Reagents, and Antibodies

Human Embryonic Kidney (HEK) 293T cells were obtained from American Type Culture Collection (ATCC) and were cultured in DMEM supplemented with fetal bovine serum (10%) and penicillin/streptomycin (1%) in 5% CO_2_ at 37°C. Poly(I:C) (HMW) was purchased from Invivogen. MG132 and anti-FLAG M2 antibody were from Sigma-Aldrich. Other antibodies used in this study were GFP, C9 tag/rhodopsin (Santa Cruz Biotechnology); BiP, XBP-1s, IRF3, p65, STAT1 (Cell Signaling Technology); and GAPDH (Proteintech).

### Plasmids

Vectors encoding Flag RIG-I, Flag MAVS, Flag TBK1, Flag IKKi, Flag IRF3, Flag IRF7, IFN-β luciferase, ISRE luciferase, NF-ĸB luciferase, and Renilla luciferase were generously provided by Dr. Genhong Cheng (University of California, Los Angeles). C9 SARS-CoV-2-Spike (alpha variant, B.1.1.7, GenBank accession number QHD43416.1) was a gift from Dr. Fang Li (Addgene plasmid #145032) and UPRE luciferase was a gift from Dr. Seiichi Oyadomari (Addgene plasmid #101788).

### Transfections and Luciferase Reporter Assays

HEK 293T cells were transfected using polyethylenimine (PEI, 1 mg/mL) (Polysciences, Inc.). Reporter assays were performed using a dual luciferase assay kit (Promega) 18-24 h after transfection with 100 ng firefly luciferase and 10 ng renilla luciferase plasmids co-transfected with indicated plasmids. Luciferase values were quantified on a luminometer (Berthold) and results for firefly luciferase activity were normalized to *renilla* luciferase activity.

### RNA Isolation and Quantitative PCR

RNA was isolated using TRIzol reagent (Invitrogen) and converted to cDNA using qScript (Quantabio). Quantitative PCR (Q-PCR) was performed using PerfeCTa SYBR green (Quantabio) in a CFX96 thermocycler (Bio-Rad). Transcript abundance was first normalized to that of mRNA encoding the ribosomal protein 36B4, then normalized against values for unstimulated controls calculated *via* the 2^-ΔΔCt^ method. Primer sequences are as follows: IFNB1 Fwd TGTGGCAATTGAATGGGAGGCTTGA, Rev CGGCGTCCTCCTTCTGGAACTG; CCL5 Fwd CGCTGTCATCCTCATTGCTA, Rev GGGTGACAAAGACGACTGCT; CXCL10 Fwd ATGAATCAAACTGCGATTCCTGATTTGCTGC, Rev TTAAGGAGATCTTTTAGCCATTTCCTTGC; IFIT2 Fwd TGCAACCTACTGGCCTATCTA, Rev CAGGTGACCAGACTTCTGATT; TNF Fwd GTGGCTGAGTCTGGTATATGGG, Rev GTGCTTTCCGTGATGAGAACC; SeV M Fwd GTGATTTGGGCGGCATCT, Rev GATGGCCGGTTGGAACAC; RPLP0/36B4 Fwd TCGAACACCTGCTGGATGAC, Rev CCACGCTGCTGAACATGCT.

### Virus Infections and Imaging

VSV (Indiana Serotype) expressing GFP and SeV has been described elsewhere (Parvatiyar et al., 2018). HEK 293T cells transfected with control vector or C9 Spike were infected with VSV-GFP or SeV (MOI 0.01) for 16 h and subsequently live imaged for VSV-GFP expression using the Echo Revolve fluorescent microscope, harvested and analyzed *via* immunoblotting, or subjected to RNA isolation for gene expression analysis.

### Immunoblot Analysis and Co-Immunoprecipitation

For immunoblot analysis, cells were harvested in ice cold NP-40 lysis buffer (50 mM Tris-Cl pH 7.4, 150 mM NaCl, 1 mM EDTA, 1% NP-40) supplemented with complete protease inhibitors (Roche) as described previously (Parvatiyar et al., 2012). For immunoprecipitation experiments, pre-cleared lysates from transfected cells were incubated overnight with anti-FLAG M2 or C9 antibodies at 4°C followed by the addition of protein A agarose beads (Roche) for 4 hours at 4°C. Captured protein complexes were washed three times with NP-40 lysis buffer containing 250 mM NaCl and then eluted with 2X Laemmli sample buffer (Bio-Rad) containing β-mercaptoethanol. Samples were boiled at 95°C for 5 minutes followed by SDS PAGE and immunoblotting. Proteins were detected *via* enhanced chemiluminescence (Pierce).

### Statistical Analysis

Quantitative data are expressed as mean -fold increase ± S.E. relative to control levels from a representative experiment performed 2-3 times in duplicate or triplicate. An *asterisk* indicates a *p* value of <0.05 as determined by a two-tailed Student’s *t*-test. Quantification of immunoblot images were performed using Image J software.

## Data Availability Statement

The raw data supporting the conclusions of this article will be made available by the authors, without undue reservation.

## Author Contributions

KP conceptualized the project and supervised the research. RF, TC, and KP performed the experiments and analyzed the data. KP prepared the manuscript. All authors contributed to the article and approved the submitted version.

## Funding

This work was supported by the Louisiana Board of Regents Support Fund LEQSF(2021-24)-RD-A-33 and Tulane SOM pilot funds awarded to KP.

## Conflict of Interest

The authors declare that the research was conducted in the absence of any commercial or financial relationships that could be construed as a potential conflict of interest.

## Publisher’s Note

All claims expressed in this article are solely those of the authors and do not necessarily represent those of their affiliated organizations, or those of the publisher, the editors and the reviewers. Any product that may be evaluated in this article, or claim that may be made by its manufacturer, is not guaranteed or endorsed by the publisher.
